# Paracetamol serum concentrations in preterm infants treated with paracetamol intravenously: a case series

**DOI:** 10.1186/1752-1947-6-1

**Published:** 2012-01-04

**Authors:** Christ-jan JLM van Ganzewinkel, Thilo Mohns, Richard A van Lingen, Luc JJ Derijks, Peter Andriessen

**Affiliations:** 1 Department of Pediatrics, Division of Neonatology, Máxima Medical Centre, De Run 4600, 5504 DB Veldhoven, The Netherlands; 2 Princess Amalia Department of Pediatrics, Division of Neonatology, PO Box 10400, 8000 GK Zwolle, The Netherlands; 3 Department of Clinical Pharmacy, Máxima Medical Center, De Run 4600, 5500 DB Veldhoven, The Netherlands

## Abstract

**Introduction:**

Until now, studies on paracetamol given intravenously have mainly been performed with the pro-drug propacetamol or with paracetamol in preterm babies above 32 weeks of gestation. Studies in these babies indicate that intravenous paracetamol is tolerated well, however studies on the efficacy of intravenous paracetamol are lacking. There are no pharmacokinetic data on the administration of multiple doses of paracetamol in preterm babies with a gestational age below 32 weeks.

**Case presentation:**

We present a case series of nine Caucasian preterm babies, six boys and three girls, with a mean gestational age of 28.6 weeks (range 25.9 to 31.6 weeks). Case one, a girl with a gestational age of 25 weeks and six days, presented with necrotizing enterocolitis. In the second case, a female baby with a gestational age of 26 weeks and two days presented with hematoma. In case three, a female baby with a gestation of 26 weeks and one day developed intraventricular hemorrhage. In case four, a male baby with a gestational age of 31 weeks and four days presented with pain after vacuum delivery. Case five, a female baby born after a gestation of 29 weeks and six days presented with hematoma. In case six, a male baby with a gestation of 30 weeks and six days presented with hematoma. In case seven, a male baby, born with a gestational age of 30 weeks and six days, presented with caput succedaneum and hematoma. In case eight, a male baby, born after a gestation of 28 weeks and four days, developed abdominal distention. Case nine, a female baby, born with a gestational age of 27 weeks and three days presented with hematoma. These babies were treated with intravenous paracetamol 15 mg/kg every six hours. Serum concentrations and aspartate transaminase were determined after prolonged administration. Pain scores were assessed using the Premature Infant Pain Profile.

**Conclusion:**

Paracetamol serum concentrations ranged from 8 to 64 mg/L after eight to 12 doses of intravenous paracetamol. Adequate analgesia was obtained in seven babies. During paracetamol therapy the median serum level of aspartate transaminase was 20 U/L (range 12 to 186 U/L). This case series indicates that prolonged intravenous administration of paracetamol in preterm babies with a gestational age of less than 32 weeks is tolerated well in the first days after birth. However, in the absence of proper pharmacokinetic data in this age group we cannot advocate the use of paracetamol intravenously.

## Introduction

Pain management in newborns is limited by the availability of only a few analgesics. The use of opiates in newborns is limited because of potential clinical side effects. As an alternative to opiates, paracetamol is a well-known analgesic in children without significant side effects. There are only limited data on the use of paracetamol in the newborn. The first drafts of the evidence-based guideline regarding pain management in children of the Dutch Pediatric Society supported the intravenous administration of paracetamol (15 mg/kg every six hours) in babies. In advance of the guideline we introduced intravenous administration of paracetamol to preterm babies in our neonatal intensive care unit to reduce the use of opiates. As a safety precaution we determined serum levels of paracetamol and aspartate transaminase in babies with intravenous paracetamol.

After the release of the final version of the nationwide evidence-based guideline on pain assessment and management in children, it became clear that the guideline restricted intravenous administration of paracetamol to term babies after the first month [1]. After the release of the final guideline we discontinued the local policy of intravenous administration of paracetamol in preterm babies. The Institutional Review Board/Independent Ethics Committee was informed afterwards and concluded that the presented data were obtained legally according the Dutch Law on Medical Research with Humans (WMO).

Though the case series of nine is achieved in an unusual manner, we consider the data on paracetamol levels in preterm babies below 32 weeks of gestation as relevant information for future clinical studies.

## Case presentations

### Case one

A Caucasian female baby was admitted to our NICU after a gestation of 25 weeks and six days. Although delivery started in the hospital the one minute Apgar score is not available because no health care provider was present at the time of birth. Her five minute Apgar score was six and her birth weight was 890 grams (p50-75). During the third week of life she developed necrotizing enterocolitis grade one according to Bell's criteria. She received 15 mg/kg intravenous paracetamol every six hours, with a total of four doses. Co-medications were antibiotics and ranitidine. Pain score, as measured with the Premature Infant Pain Profile (PIPP) decreased from 10 to eight (12 or more reflects moderate to severe pain). After 24 hours paracetamol was discontinued because of low PIPP scores. The paracetamol serum level determined four hours after the last dose was 24 mg/L.

### Case two

A Caucasian female baby, born with a gestational age of 26 weeks and two days, was admitted with respiratory failure to our NICU. She was intubated shortly after delivery. Apgar scores were one and five after one and five minutes respectively. Birth weight was 680 grams (p5-10). Because of hematoma she received 15 mg/kg intravenous paracetamol every six hours. Therapy was started four hours after birth. She received a total of six doses. Pain scores decreased from 10 to nine only. Co-medications were antibiotics and caffeine. The paracetamol serum level, which was determined three hours after the last dose, was 29 mg/L.

### Case three

A Caucasian female baby was admitted to our NICU after a gestation of 26 weeks and one day. Shortly after birth she developed respiratory failure and was intubated. Apgar scores were one and five after one and five minutes, respectively. Her birth weight was 800 grams (p25-50). She developed a grade three intra-ventricular hemorrhage for which morphine was started. Further co-medications were antibiotics. In an attempt to stop morphine, paracetamol was started, in a dose of 15 mg/kg every six hours. Pain scores were below six during morphine and remained so during paracetamol mono-therapy. She received six doses of paracetamol and the serum level was determined 20 hours after the last dose. The serum level was 12 mg/L.

### Case four

A Caucasian male baby, born with a gestational age of 31 weeks and four days, was admitted to our NICU after vacuum delivery. Apgar scores were eight and nine after one and five minutes, respectively. His birth weight was 1600 grams (p25-50). He received 15 mg/kg of intravenous paracetamol every six hours for a total of eight doses. Pain scores decreased from 14 before start of therapy to nine during therapy. The paracetamol serum level was determined 10 hours after the last dose and was 25 mg/L.

### Case five

A Caucasian female baby, born after a gestation of 29 weeks and six days, was admitted with hematoma due to traumatic birth and breech delivery to our NICU. After birth she received cardiopulmonary resuscitation because of apnea and bradycardia. Apgar scores were one and six after one and five minutes, respectively. Her birth weight was 1300 grams (p25). She was diagnosed with hematoma and received 15 mg/kg of intravenous paracetamol every six hours starting five hours after birth. She received a total of nine doses of paracetamol. One hour after the last dose her paracetamol serum level was 46 mg/L. Thirty hours later the serum level was determined again and was < 5 mg/L. Co-medications consisted of antibiotics and caffeine. Pain scores decreased from 16 before start of paracetamol to nine during therapy.

### Case six

A male Caucasian baby was admitted to our NICU after a gestation of 30 weeks and six days. Birth took place in a peripheral hospital and was complicated by breech presentation and forceps delivery. Apgar scores were two and seven after one and five minutes, respectively. Birth weight was 1480 grams (p25). In the first hours of life he developed respiratory failure and was intubated. The baby showed extensive hematoma for which 15 mg/kg of intravenous paracetamol every six hours was started. He received a total of 10 doses. Three hours after the last dose his paracetamol serum level was 64 mg/L. Co-medications were antibiotics and caffeine. Pain scores decreased from 10 before therapy to six during therapy.

### Case seven

A Caucasian male baby, born with a gestational age of 30 weeks and six days, was admitted to our NICU after an uneventful preterm delivery. The Apgar scores were nine and 10 after one and five minutes, respectively. His birth weight was 1755 gram (p50-75). He was diagnosed with caput succedaneum and also had a small hematoma on one of the upper limbs. Due to high pain scores he received 15 mg/kg of intravenous paracetamol every six hours starting two hours after birth. He received a total of 11 doses of paracetamol. His serum paracetamol level was 37 mg/L four hours after the last does. He received no co-medication. Pain scores decreased from 14 before start of paracetamol to seven during analgesic therapy.

### Case eight

A Caucasian male baby, born after a gestation of 28 weeks and four days, was admitted with respiratory failure due to respiratory distress syndrome to our NICU. Apgar scores were four and eight after one and five minutes, respectively. His birth weight was 860 grams (p25-50). He developed severe abdominal distention on the second day of life and received 15 mg/kg of intravenous paracetamol every six hours for a total of 14 doses. There were no radiological signs of necrotizing enterocolitis and his condition improved over the next few days. Co-medications were antibiotics and caffeine. Five hours after the last dose his paracetamol serum level was 8 mg/L. Pain scores decreased from 14 before starting paracetamol to three during therapy.

### Case nine

A Caucasian female baby, born with a gestational age of 27 weeks and three days was admitted to our NICU after preterm rupture of membranes and an uncomplicated delivery. Apgar scores were six and nine after one and five minutes, respectively. Her birth weight was 990 gram (p50). Due to hematoma and subsequent high pain scores (14) she received 15 mg/kg of intravenous paracetamol every six hours. She received a total of 17 doses. Due to inadequate analgesic effect 10 μg/kg/hour morphine was started during paracetamol therapy. Her paracetamol serum level, determined seven hours after the last dose, was 61 mg/L.

Table 1 summarizes the clinical data of the nine babies.

**Table 1:** Clinical data of the case series

Cases	Gestational age (wk)	Birth weight (g)	Duration of therapy (hr)	Start therapy (hr after birth)	Interval last dose-blood sample (hr)	Serum concentration (mg/L)
1	25.9	890	24	408	4	24
2	26.4	680	36	4	3	29
3	26.1	800	36	72	20	12
4	31.6	1600	48	1	10	25
5	29.9	1300	54	5	1	46
6	30.9	1480	60	1	3	64
7	30.9	1755	66	2	4	37
8	28.6	860	84	37	5	8
9	27.4	990	102	1	7	61

**Figure 1:**
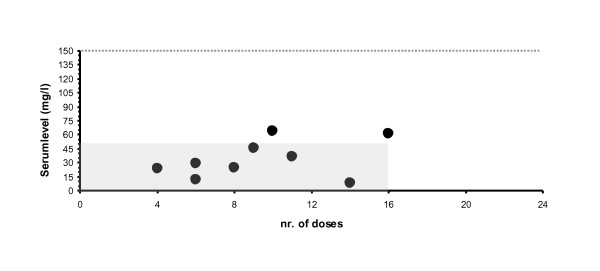
**Paracetamol serum levels in relation to number of doses**. In seven babies the serum levels of paracetamol (the black dots) are < 50 mg/L (grey area), the upper margin value found by Palmer for babies > 32 weeks of gestation [2]. The highest serum concentration (64 mg/L) was far below 150 mg/L (indicated by the dotted grey horizontal line), which has been reported as a toxic value in children [3].

Figure 1 shows the paracetamol serum concentrations of the nine babies, related to the number of doses. In seven babies the serum levels of paracetamol (the black dots) are < 50 mg/l (grey area), the upper margin value found by Palmer for babies > 32 weeks of gestation [2]. The highest serum concentration (64 mg/l) was far below 150 mg/l (indicated by the dotted grey horizontal line), which has been reported as a toxic value in children [3].

## Discussion

We administered intravenous paracetamol in a dose not supported by literature. The dose we used in preterm babies of less than 32 weeks gestation is being used in term babies, and is not a result of miscalculation due to the differences in formulations of propacetamol and paracetamol [4].

Until now, most studies on intravenous paracetamol have been performed with propacetamol in preterm babies above 32 weeks of gestation. Propacetamol is a pro-drug of paracetamol and is hydrolyzed by plasma esterases after intravenous administration such that 1 g of propacetamol is hydrolyzed to 0.5 g of paracetamol [4-6]. To our knowledge this is the first report of paracetamol concentration data in preterm babies below 32 weeks of gestation, in whom multiple dose intravenous paracetamol (Perfalgan^®^) was administered for non-surgical analgesia in the first hours after birth.

This case series indicates that in preterm babies below 32 weeks intravenous paracetamol is tolerated well. In seven babies we found serum concentrations below 50 mg/l, the upper margin value reported by Palmer [2]. In two subjects serum values were around 60 mg/l. During paracetamol therapy we found no indications for liver failure.

Although the therapeutic window for paracetamol in children is assumed to be 10-20 mg/l, there is no consensus on dosage regimens for intravenous administration of paracetamol in babies [7]. Allegaert, using propacetamol, suggests a maintenance dose of 20 mg/kg every 12 hours for babies below 31 weeks gestational age after a loading dose of 30 mg/kg propacetamol [4]. Using this dose, Allegaert was not able to show significant analgesic effect [5]. However, with a maintenance dose of 12.5 mg/kg every six hours Allegaert showed analgesic effects [6]. Autret suggests a maximum of 7.5 mg/kg every 6 hours after a loading dose of 15 mg/kg propacetamol in newborns for antipyretic effects [8]. Autret did not study the analgesic effect. In term newborns, de la Pintière describes a maintenance dose of intravenous propacetamol of 120 mg/kg/day, equivalent to paracetamol 60 mg/kg/day [9].

Limited data is available concerning the pharmacokinetics of propacetamol and paracetamol [2,5,6,10]. Both Allegaert and Palmer found serum levels of paracetamol between < 6 and 50 mg/l, after a single dose of propacetamol and multiple doses of intravenous paracetamol, respectively. Note that Palmer included preterm babies above 32 weeks of gestation [2]. In a letter to the editor, Bartocci *et al *report their Stockholm experience of postoperative analgesia with intravenous morphine and paracetamol (maintenance dose 7.5 mg/kg every eight hours) in newborns with a postconceptional age between 25 and 42 weeks [11]. From the letter, however, it is unclear at what postnatal age paracetamol is given and no paracetamol concentration data are shown.

Several cases report accidentally given overdoses of propacetamol or paracetamol. Two doses of approximately 300 mg/kg propacetamol (equivalent to 150 mg/kg paracetamol) at a 6 hour interval given to a term baby, resulted in a serum level of 166 mg/l without signs or symptoms of liver failure [9]. Two babies born prematurely after maternal overdose of paracetamol had serum concentrations of 76 and 260 mg/l respectively, without apparent adverse effects [12,13]. A paracetamol overdose in a preterm baby resulted in a serum concentration of 121 mg/l [14].

Recently, Bristol-Myers Squibb Pharmaceuticals Ltd issued a letter with drug safety information concerning accidental overdose in 23 world wide cases. All were babies younger than one year, one of whom died. Scope of the letter was a raising concern on the possible confusion in prescribing ml/kg instead of mg/kg, leading to a tenfold overdose [15]. The letter does not provide information on serum levels or liver functions in these cases.

## Conclusion

This case series is not a formal pharmacokinetic study. Obviously, the small sample size and the single serum concentration limit a pharmacokinetic interpretation of paracetamol therapy in preterm babies. Still, this case series of nine very preterm babies indicates that paracetamol administration in a maintenance dose of 15 mg/kg/day every six hours results in paracetamol concentrations that are in the range of others [2,5,10]. It suggests that intravenous paracetamol is tolerated well in the first hours after birth in very preterm babies. However, since proper pharmacokinetic data in this age group is still lacking, we cannot advocate the use of paracetamol intravenously based on our observations. It is obvious that future studies should target determination of dosing regimens to achieve maximum analgesic effect (efficacy) without adverse effects (tolerance) in newborns in the first four weeks after birth.

### Consent

Written informed consent was obtained from the parents of the patients for publication of this case report. A copy of the written consent is available for review by the Editor-in-Chief of this journal.

## Competing interests

The authors declare that they have no competing interests.

## Authors' contributions

CG was responsible for data collection, analysis and drafted the paper. PA analyzed the data, reviewed the paper, and was a major contributor in writing the manuscript. TM contributed to data collection and reviewed the paper. LD analyzed the data and reviewed the paper. RL reviewed the paper. All authors read and approved the final manuscript.
